# Supplementary material for: Content-rich biological network constructed by mining PubMed abstracts
Source: BMC Bioinformatics. 2004 Oct 8;5:147. doi: 10.1186/1471-2105-5-147 (PMC528731; doi:10.1186/1471-2105-5-147)
Supplement: Additional File 5 — The original Chilibot query results of the term "long-term potentiation (LTP)" and 22 other terms, limiting the latest references analyzed to the years 1990, 1995, 2000, and 2004. [file 1471-2105-5-147-S5.bz2 › chilibotAdditionalFile5/ltp2004/html/LTP_ARC.html]

 


 **LTP** and **ARC** 
  
Found 12 abstracts in PubMed,  **12 abstracts were retrieved and analyzed**.  


---

 Search Google  |
 PDF files only 
|  EDU domain only 

---

**Interactive relationship** (e.g. stimulation, inhibition, etc)

- Here we report that arg3.1  **arc**  is robustly induced with  **LTP**  stimulation even at intensities that are not sufficient to activate c fos expression.  Ref: 11466419 J Neurosci, 2001
- Our studies show that disruption of  **Arc**  protein expression impairs the maintenance phase of  **LTP**  without affecting its induction and impairs consolidation of LTM for spatial water task training without affecting task acquisition or short term memory.  Ref: 10818134 J Neurosci, 2000
- These results establish the existence of subfield specific transcriptional responses to  **LTP**  inducing stimulation in the hippocampus of the intact animal, and demonstrate that in area CA1 enhanced transcription of Zif268,  **Arc**  and Homer is not required for the induction of late  **LTP** .  Ref: 11264669 Eur J Neurosci, 2001
- **Arc**  is strongly induced by patterns of synaptic activity that also induce long term potentiation  [ **LTP** ] , and  **Arc**  mRNA is then rapidly delivered into dendrites after episodes of neuronal activation.  Ref: 11416188 Proc Natl Acad Sci U S A, 2001
- Despite the inhibition in total protein synthesis, the level of the immediate early gene protein  **Arc**  activity regulated cytoskeleton associated protein increased at 1 hour after chemical long term potentiation  [ **LTP** ]  induction.  Ref: 12573716 Neuroscience, 2003
- In granule cells of the dentate gyrus,  **LTP**  induced in vivo by tetanic stimulation of the perforant path was followed by strong induction of the immediate early genes IEGs Zif268,  **Arc**  and Homer.  Ref: 11264669 Eur J Neurosci, 2001
- We have found that selective translation of  **Arc**  in a synaptoneurosomal preparation is induced by the brain derived neurotrophic factor, a neurotrophin that is released during high frequency stimulation patterns used to elicit  **LTP** .  Ref: 11842217 Proc Natl Acad Sci U S A, 2002
- mRNA for the activity regulated cytoskeletal protein,  **Arc** , is transcribed during  **LTP**  and learning, and disruption of its translation gives rise to deficits in both.  Ref: 11842217 Proc Natl Acad Sci U S A, 2002
- Formation of stable, late phase HFS  **LTP**  requires mRNA synthesis and is coupled to upregulation of the immediate early gene activity regulated cytoskeleton associated protein  **Arc** .  Ref: 12196567 J Neurosci, 2002
- Inhibition of activity dependent  **arc**  protein expression in the rat hippocampus impairs the maintenance of long term potentiation  [ **LTP** ]  and the consolidation of long term memory.  Ref: 10818134 J Neurosci, 2000

**Parallel relationship** (e.g. studied together, co-existance, homology, etc.)

- U0126 blocked  **Arc**  upregulation in parallel with BDNF  **LTP** .  Ref: 11880483 J Neurosci, 2002
- At the age when these animals developed cognitive dysfunction, they had reduced mRNA expression of several genes essential for long term potentiation  [ **LTP** ]  and memory formation  **Arc** , Zif268, NR2B, GluR1, Homer 1a, Nur77 TR3.  Ref: 12832546 J Neurosci, 2003
- In contrast, we were unable to detect modulation of the IEGs Zif268,  **Arc** , Homer and HB GAM following induction of  **LTP**  by high frequency stimulation of the commissural projection to CA1 pyramidal cells in vivo.  Ref: 11264669 Eur J Neurosci, 2001
- Local infusion of the transcription inhibitor actinomycin D ACD 1 hr before or immediately before BDNF infusion inhibited BDNF  **LTP**  and upregulation of  **Arc**  protein expression.  Ref: 12196567 J Neurosci, 2002
- **Arc**  Activity regulated, cytoskeletal associated protein is an effector immediate early gene implicated in  **LTP**  and other forms of neuroplasticity.  Ref: 14580947 Neuroscience, 2003
- When  **LTP**  was induced in vitro neither CA1 pyramidal cells nor granule cells showed an increase in Zif268,  **Arc**  or Homer mRNA.  Ref: 11264669 Eur J Neurosci, 2001
- Previous studies demonstrate a role for  **Arc**  in the maintenance of long term potentiation  [ **LTP** ]  and in memory consolidation processes and indicate a role for H1a in modifying glutamatergic signaling pathways.  Ref: 12451105 J Neurosci, 2002
- We used intrahippocampal infusions of antisense oligodeoxynucleotides to inhibit  **Arc**  protein expression and examined the effect of this treatment on both  **LTP**  and spatial learning.  Ref: 10818134 J Neurosci, 2000
- We will review findings from other laboratories that both  **LTP**  and memory are disrupted when  **Arc**  induction is blocked, and we will discuss the possibility that  **Arc**  protein may play a key role in activity dependent synaptic modification.  Ref: 12559831 Neurobiol Learn Mem, 2002
